# Motor Imagery EEG Classification Based on Decision Tree Framework and Riemannian Geometry

**DOI:** 10.1155/2019/5627156

**Published:** 2019-01-21

**Authors:** Shan Guan, Kai Zhao, Shuning Yang

**Affiliations:** School of Mechanical Engineering, Northeast Electric Power University, 132012 Jilin, China

## Abstract

This paper proposes a novel classification framework and a novel data reduction method to distinguish multiclass motor imagery (MI) electroencephalography (EEG) for brain computer interface (BCI) based on the manifold of covariance matrices in a Riemannian perspective. For method 1, a subject-specific decision tree (SSDT) framework with filter geodesic minimum distance to Riemannian mean (FGMDRM) is designed to identify MI tasks and reduce the classification error in the nonseparable region of FGMDRM. Method 2 includes a feature extraction algorithm and a classification algorithm. The feature extraction algorithm combines semisupervised joint mutual information (*semi*-JMI) with general discriminate analysis (GDA), namely, SJGDA, to reduce the dimension of vectors in the Riemannian tangent plane. And the classification algorithm replaces the FGMDRM in method 1 with k-nearest neighbor (KNN), named SSDT-KNN. By applying method 2 on BCI competition IV dataset 2a, the kappa value has been improved from 0.57 to 0.607 compared to the winner of dataset 2a. And method 2 also obtains high recognition rate on the other two datasets.

## 1. Introduction

Brain computer interface (BCI) based on motor imagery (MI) is used to analyze human intention by electroencephalogram (EEG) signals generated by human brain electrophysiological activity [1, 2]. Based on BCI technology, exoskeletons can be used to help people with physical disabilities regain their motor ability, and BCI also has wide applications in smart home, entertainment, military, and other fields [3–6].

Common spatial pattern (CSP) is widely used in motor imagery to extract EEG features [7]. CSP has excellent performance in two classification tasks, but the drawback is that it needs a lot of electrodes [8].

Despite its short history, the use of the Riemannian geometry in BCI decoding is currently attracting increasing attention [9–13]. Covariance matrices lie in the space of symmetric positive definite (SPD) matrices, which can be formulated as a Riemannian manifold [14]. In the BCI field, the connections of the CSP algorithm and the tools of information geometry have been investigated, considering several divergence functions in alternative to the Riemannian distance [15–18]. Barachant et al. proposed a simple data augmentation approach for improving the performance of the Riemannian mean distance to mean (MDM) algorithm [13]. Kumar et al. propose a single band CSP framework for MI-BCI that utilizes the concept of tangent space mapping in the manifold of covariance matrices, and the proposed method obtains good results when compared to other competing methods [19]. A hierarchical MDM classifier for multiclass problem has been tested in [20].

Advanced classifiers based on the tangent space on the Riemannian manifold of positive matrices are also receiving increasing attention. Barachant et al. map the covariance matrices in the tangent space and apply feature selection and linear discriminate analysis (LDA) in the tangent space [10]. For the application of the classifier in the tangent space, the problem is that the curse of dimensionality. Traditional data dimensionality reduction methods include two categories: linear dimensionality reduction (LDR) and nonlinear dimensionality reduction (NLDR). Since most of the actual data are nonlinear, NLDR techniques such as locally linear embedding (LLE) [21], isometric mapping (ISOMAP) [22], maximum variance unfolding (MVU) [23], and t-distributed stochastic neighbor embedding (t-SNE) [24, 25] are used to tackle problems widely. Lee et al. used discrete wavelet transform (DWT) and continuous wavelet transform (CWT) to extract features of MI tasks, and Gaussian mixture model (GMM) was used to construct GMM supervectors; this method accelerates the speed of training and improves the accuracy of motor imagery [26]. Sadatnejad et al. propose a new kernel to preserve the topology of data points in the feature space, and the proposed kernel is strong, particularly in the cases where data points have a complex and nonlinear separable distribution [8]. Xie et al. proposed a framework for intrinsic submanifold learning from a high-dimensional Riemannian manifold; the proposed method exhibited strong robustness against a small training dataset [27].

There is still another approach for overcoming the problem of high dimensionality in SPD manifolds. And this method maps from a high-dimensional SPD manifold to a lower dimensional one while the geometry of SPD manifolds is preserved. And there are only two works of this way. Davoudi et al. [14] proposed distance preservation to local mean (DPLM) as dimensionality reduction technique, combined with FGMDM, the best performance of this article in terms of kappa value is 0.60. Harandi et al. [28] learned a mapping that maximizes the geodesic distances between interclass and simultaneously minimizes the distances between intraclass, and it is done via an optimization on Grassmann manifolds.

In this paper, we proposed a novel SSDT-FGMDRM and SSDT-KNN for the classification of multiclass MI tasks by designing a simple yet efficient subject-specific decision tree framework. Method 1 contains SSDT-FGMDRM to improve the performance of FGMDRM. For each individual, method 1 first separates the two most discriminative classes from the group. Furthermore, the remaining categories including the misclassification samples of the previous nodes are reclassified in the last node. Method 2 contains SSDT-KNN and a NLDR method named SJGDA. SJGDA combines the advantage of *semi*-JMI and GDA, and method 2 performed well on different datasets. The aims of this article are as follows:

1. To verify the effectiveness of the proposed SSDT framework through dataset 1
2. To verify the superiority of SJGDA in feature extraction, compared with *semi*-JMI and GDA
3. To validate the generalization ability of method 2 through different datasets, in this paper

The rest of the paper is organized as follows: Section 2 introduced the mathematical preliminaries of the Riemannian geometry.  Section 3 discussed the proposed methods in detail. Three datasets are introduced in Section 4. The results of our work are discussed in Section 5. And in Section 6, we compared our methods with the state of the art. This paper concludes in Section 7.

## 2. Geometry of SPD Matrices

Let *X*_*i*_ represent a short segment of continuous EEG signals, and *X*_*i*_ can be denoted as follows:

(1) $$\begin{array}{c} X_{\text{i}}=\left[X_{t+T_{i}}\cdots X_{t+T_{i}+T_{\text{s}}-1}\right]\in R^{n\times T_{\text{s}}}, \end{array}$$

where *X*_*i*_ corresponds to the *i*th trail of imaged movement starting at time *t* = *T*_*i*_. *T*_s_ denotes the number of sampled points of the selected segment.

For the *i*th trail, the spatial covariance matrix (SCM) *P*_i_ ∈ *ℝ*^*n*×*n*^ can be calculated as follows:

(2) $$\begin{array}{c} P_{i}=\frac{1}{T_{\text{s}}-1}X_{i}X_{i}^{T}. \end{array}$$

Based on the SCM, there are two ways to classify MI tasks in the Riemannian manifold.

### 2.1. Filter Geodesic Minimum Distance to the Riemannian Mean

The Riemannian distance between two SPD matrices *P*_1_ and *P*_2_ in *P*(*n*) is given by [29]

(3) $$\begin{array}{c} \delta_{R}\left(P_{1},P_{2}\right)={\left\|\text{log}\left(P_{1}^{-1}P_{2}\right)\right\|}_{F}={\left[\overset{n}{\underset{i=1}{\sum }}{\text{log}}^{2}\;\lambda_{i}\right]}^{1/2}. \end{array}$$

Given *m* SPD matrices *P*_1_, … , *P*_*m*_, the geometric mean in the Riemannian sense is defined as

(4) $$\begin{array}{c} ℑ\left(P_{1},\ldots ,P_{m}\right)=\underset{P\in P\left(n\right)}{\arg\text{ min}}\overset{m}{\underset{i=1}{\sum }}\delta_{R}^{2}\left(P,P_{i}\right). \end{array}$$

For algorithm mean Riemannian distance to Riemannian mean (MDRM), we compute the Riemannian distance between unknown class *P* to the Riemannian mean point of each class and classify the unknown class into categories corresponding to the shortest distance. Inspired by the principal geodesics analysis (PGA) method [30], the literature [31] finds a set of filters by applying an extension of Fisher linear discriminant analysis (FLDA) named Fisher geodesic discriminant analysis (FGDA). And then, apply these filters to MDRM to form filter geodesic minimum distance to Riemannian mean (FGMDRM). More details can be seen from [31].

### 2.2. Tangent Space Mapping

**Figure 1:**
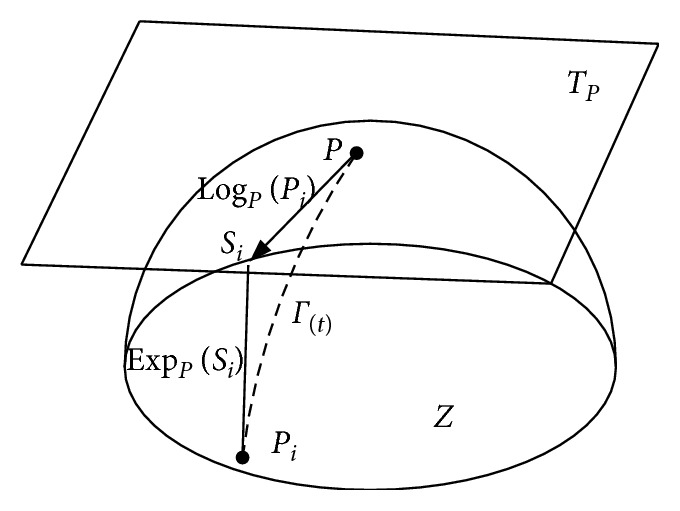
The tangent space at point *P*, and the geodesic *Γ*(t) between *P* and *P*_*i*_.

As shown in Figure 1, the SPD matrix of *P* is denoted by a differentiable Riemannian manifold *Z*. Each tangent vector *S*_*i*_ can be seen as the derivative at *t* = 0 of the geodesic *Γ*(*t*) between *P* and the exponential mapping *P*_*i*_ = EXP_*P*_(*S*_*i*_), defined as follows:

(5) $$\begin{array}{c} E{\text{xp}}_{P}\left(S_{i}\right)=P_{i}=P^{1/2}\exp\left(P^{-1/2}S_{i}P^{-1/2}\right)P^{1/2}. \end{array}$$

The inverse mapping is given by the logarithmic mapping and can be defined as follows:

(6) $$\begin{array}{c} {\text{log}}_{P}\left(P_{i}\right)=S_{i}=P^{1/2}\text{ log}\left(P^{-1/2}P_{i}P^{-1/2}\right)P^{1/2}. \end{array}$$

Using the Riemannian geodesic distance, the Riemannian mean of *I* > 1 SPD matrices by

(7) $$\begin{array}{c} ℑ\left(P_{1},\ldots ,P_{I}\right)=\underset{P\in P\left(n\right)}{\arg\text{ min}}\overset{I}{\underset{i=1}{\sum }}\delta_{\text{R}}^{2}\left(P,P_{i}\right). \end{array}$$

Using the tangent space located at the geometric mean of the whole set trials, *P*_*ℑ*_=*ℑ*(*P*_*i*_, *i*=1 … *I*), and then, each SCM *P*_*i*_ is mapped into this tangent space, to yield the set of *m* *=* *n*(*n* + 1)/2 dimensional vectors:

(8) $$\begin{array}{c} S_{i}=\text{upper}\left(P_{ℑ}^{-1/2}\text{ log}\left(P_{i}\right)P_{ℑ}^{-1/2}\right). \end{array}$$

Many efficient classification algorithms can be implemented in the Riemannian space [10].

## 3. Methods

### 3.1. Subject-Specific Decision Tree Framework

**Figure 2:**
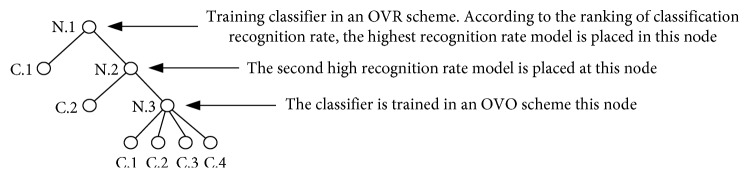
SSDT based on the best separating principle for four class. N. *i* represents node *i*, and C. *i* represents class *i*.

Decision tree is a common machine learning method. Each node of decision tree can be defined as a rule. Guo and Gelfand [32] proposed classification trees with neural network, and this method embeds multilayer neural networks directly in nodes. In the decision tree, one of the most important things is to construct a proper binary tree structure; the upper nodes have the greater impact of the accuracy of the whole samples [33]. In order to solve the multiclassification problem in this paper, we constructed a subject-specific decision tree (SSDT) classification framework as shown in Figure 2 according to the best separating principle [34]. As can be seen from Figure 2, the SSDT proposed in this paper trains a different classification model at different nodes of the decision tree.

The advantages of the SSDT framework are as follows:

1. This model separates the two MI tasks (e.g., C.1 and C.2) with the highest recognition rate as far as possible
2. At the last node, we reclassify some samples to enhance the classification ability of the classifier

### 3.2. Method 1: A Direct Classification Method Based on SSDT-FGMDRM

**Figure 3:**
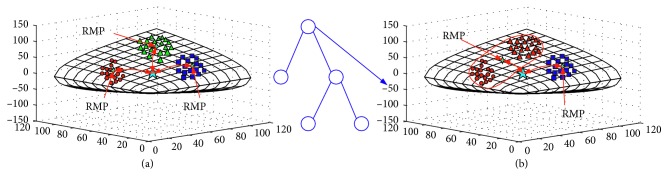
Three classification problems classified by FGMDRM (a); and a subjectspecific decision tree FGMDRM model (b).

Firstly, we point out one problem of the multiclass FGMDRM by using an example.  Figure 3 gives a three-class classification problem.  Figure 3(a) shows the classification progress by FGMDRM. We can see that three Riemannian mean points (RMPs) are located on the manifold. Since the classification criterion is decided by the distance calculated between the test point and the RMP, it caused a wrong classification.  Figure 3(b) shows the example of the classification results obtained by using the first node of the SSDT-FGMDRM framework. It can be seen that the error classification is corrected by using the decision tree framework.

**Figure 4:**
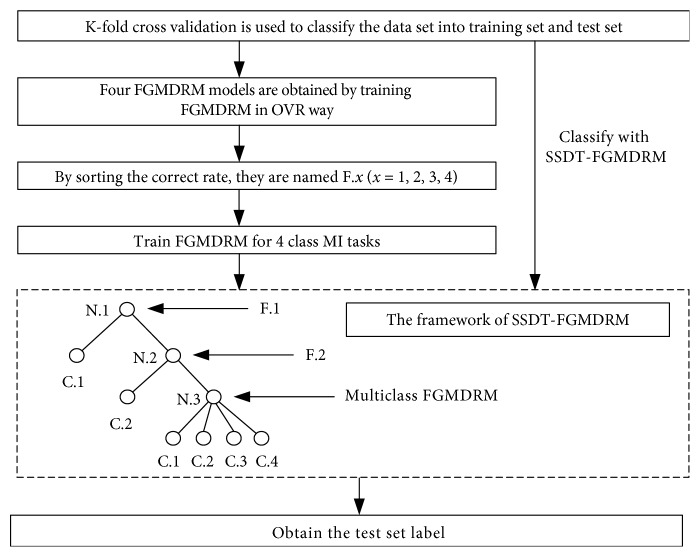
Block diagram for method 1.

Method 1 is used to classify four types of MI tasks directly. The training and testing diagram is shown in Figure 4.

### 3.3. Feature Extraction Algorithm Based on the Riemannian Tangent Space

In this paragraph, we propose a novel data reduction method which combines *semi*-JMI and GDA, namely SJGDA, to solve the dimension disaster problem after tangent space mapping.

#### 3.3.1. Semisupervised Joint Mutual Information

Semisupervised dataset *D* = *D*{*D*_L_ ∪ *D*_U_} consists of two parts, *D*_L_={x^*i*^,y^*i*^}_*i*=1_^*N*_L_^ are labelled data and *D*_U_={x^*N*_L_+*i*^}_*i*=1_^*N*_U_^ are unlabelled data. A binary random variable *S* is introduced to determine the distribution of labelled dataset and unlabelled dataset. When *s* = 1, we record the value of *y*, otherwise not. In this way, the labelled set *D*_L_ comes from the joint distribution *p*(*x*, *y*|*s* = 1), while the unlabelled set *D*_U_ comes from the distribution *p*(*x*|*s* = 0). The underlying mechanism *S* turns out to be very important for feature selection.

Feature selection method based on mutual information theory is a common feature selection method [35]. In these methods, we rank the features according to the score and select the features with higher scores. For example, by ranking the features according to their mutual information with the labels, we get the sort of correlation that is related to class labels. The characteristics of the score are defined as follows:

(9) $$\begin{array}{c} J_{\text{JMI}}\left(X_{k}\right)=\underset{X_{j}\in X_{\theta }}{\sum }\hat{I}\left(X_{k};\frac{Y}{X_{j}}\right), \end{array}$$

where *X*_*θ*_ represents the set of the features already selected and *X*_*k*_ is the feature ranked by scores. *Y* represents the label corresponding to feature *X*_*k*_.

*Semi*-JMI is a method of using a semisupervised dataset as a training set for JMI. More details can be seen from Reference [36]. In this paper, the missingness mechanism is class-prior-change semisupervised scenario (MAR-C) [37]. After feature ranking, we can obtain a feature vector as follows:

(10) $$\begin{array}{c} f=\left[f_{1},f_{2},\ldots ,f_{n}\right], \end{array}$$

where *n* is the length of the tangent vectors *S*_*i*_. Since information redundancy exists in *f*, we select the best vector length *m* (*m* < *n*) of each subject by the classification recognition rate:

(11) $$\begin{array}{c} f_{\text{SJ}}=\left[f_{1},f_{2},\ldots ,f_{m}\right]. \end{array}$$

#### 3.3.2. Generalized Discriminant Analysis

After variable selection, this paper uses generalized discriminant analysis (GDA) [38, 39], which is a nonlinear feature reduction technique based on kernels to reduce the length of the feature vectors *f*_SJ_ and their redundancies. Mapping *X* (*f*_SJ_) into a high-dimensional space *F* through a kernel function Φ:

(12) $$\begin{array}{c} \Phi :R^{d}⟶F,\\ x^{\Phi }\left|⟶\right.\left(x\right). \end{array}$$

The linear Fisher decision is performed in the *F* space, and the criterion function for its extension is

(13) $$\begin{array}{c} J\left(W^{\Phi }\right)=\text{arg}\underset{W^{\Phi }}{\text{max}}\left(\frac{\left|{\left(W^{\Phi }\right)}^{T}S_{B}^{\Phi }W^{\Phi }\right|}{\left|{\left(W^{\Phi }\right)}^{T}S_{T}^{\Phi }W^{\Phi }\right|}\right), \end{array}$$

where *W*^Φ^ ∈ *F* and *S*_B_ and *S*_W_ are between-class scatter and within-class scatter, respectively.

For the convenience of the numerical calculation, kernel functions are introduced to solve the problem:

(14) $$\begin{array}{c} k\left(x,y\right)=\left(\Phi \left(x\right)\cdot \Phi \left(y\right)\right). \end{array}$$

Gauss kernel, poly kernel, and sigmoid kernel are widely used in GDA [40]. For test data *z*, its image Φ(*z*) in *F* space projects on *W*^Φ^ is as follows:

(15) $$\begin{array}{c} \left(W_{i}^{\Phi }\cdot \Phi \left(z\right)\right)=\overset{N}{\underset{j=1}{\sum }}\alpha_{ij}\left(\Phi \left(x_{j}\right)\cdot \Phi \left(z\right)\right)=\overset{N}{\underset{j=1}{\sum }}\alpha_{ij}k\left(x_{j},z\right). \end{array}$$

This paper uses ploy kernel to reduce the dimension. After GDA, we can get a vector *f*_G_ as follows:

(16) $$\begin{array}{c} f_{\text{G}}=\left[f_{1},f_{2},\ldots ,f_{d}\right], \end{array}$$

where *d* of *f*_G_ is decided by the actual needs, and in this paper we set *d* = 1. And then, SJGDA is applied to the dataset of this paper, and the final feature vectors are constructed as follows:

(17) $$\begin{array}{c} f_{\text{SJGDA}}=\left[f_{\text{G}},f_{\text{SJ}}\right]. \end{array}$$

### 3.4. Method 2: SJGDA and Subject-Specific Decision Tree k-Nearest Neighbor

**Figure 5:**
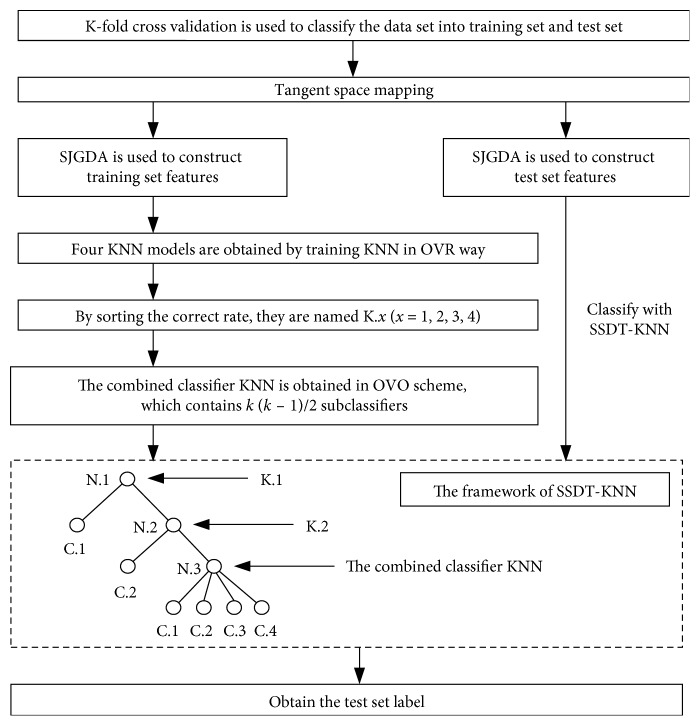
Block diagram for method 2.

Method 2 is used to classify four types of MI tasks after tangent space mapping. The training and testing diagram is shown in Figure 5.

## 4. Description of Data

### 4.1. Dataset 1

BCI competition IV dataset 2a is used to evaluate the performance of the proposed two methods [41]. Dataset 2a collects 22 channel EEG data and 3 EOG channel data. Four types of motor imagery were collected: left hand, right hand, foot, and tongue. The dataset contains nine healthy subjects and each subject has two sessions, one training session and one test session. Each session has 288 trails of MI data with 72 trails for each MI task. The EEG signals are bandpass filtered by a 5-th order Butterworth filter in the 8–30 Hz frequency band. The selection of trial period is important in MI classification; we select 2 s data (0.5 s and 2.5 s) after the cue, instructing the user to perform the MI tasks by the winner of the competition.

### 4.2. Dataset 2

BCI competition III dataset IIIa is used to evaluate the performance of method 2. BCI III dataset IIIa contains 3 subjects: K3b, K6b, and L1b, and collects 64 channel EEG data. The EEG was sampled with 250 Hz. Four types of motor imagery were collected: left hand, right hand, foot, and tongue. More details about this dataset can be seen at Reference [42].

### 4.3. Dataset 3

**Figure 6:**
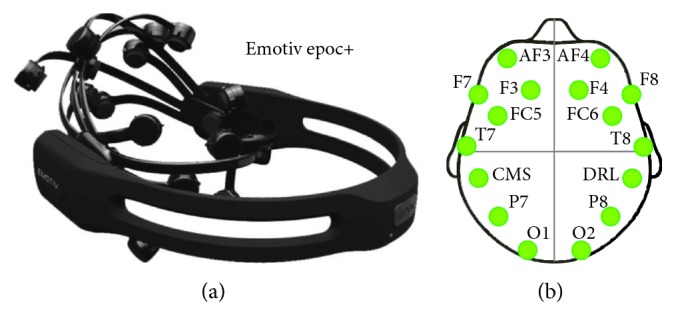
(a) Emotiv Epoc+ and (b) Emotiv 14 electrodes located over 10–20 international system positions.

**Figure 7:**
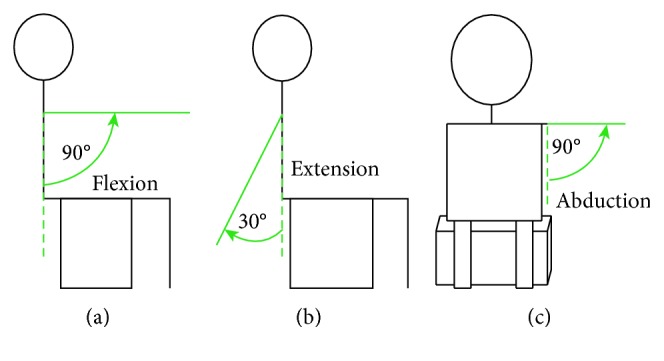
Three movements of shoulder joint: (a) flexion, (b) extension, and (c) abduction.

In our own dataset, Emotiv Epoc+ is used to collect EEG data of motor imagery. It is a portable EEG acquisition device with a sampling rate of 128 Hz. It has fourteen electrode channels (AF3, F7, F3, FC5, T7, P7, O1, O2, P8, T8, FC6, F4, F8, and AF4), two inference electrodes (CMS and DRL), and the electrode placement follows the international 10–20 standard. Equipment and the Emotiv 14 electrodes are located over 10–20 international system positions as shown in Figure 6. This experiment collected three kinds of EEG signals of one joint: imagination of shoulder flexion (F), extension (E), and abduction (A), as shown in Figure 7.

**Figure 8:**
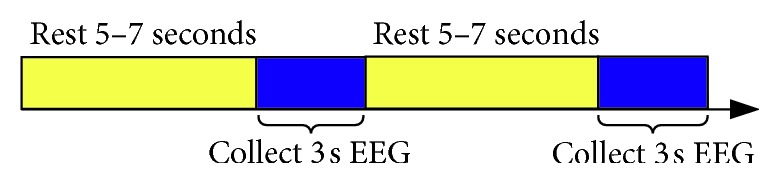
Timing for experimental process.

Seven subjects participated in this experimental study. These subjects were in good health. During the experiment, subjects were naturally placed with both hands, trying to avoid body or head movement. During the experiment, subjects carried out motor imagery under the outside cue, a single experiment collected EEG signal for 5 seconds, and then took 5–7 seconds to have rest, each action repeated acquisition 20 times. The experimental process is shown in Figure 8.

## 5. Results

### 5.1. Results of Method 1

We use SSDT-FGMDRM to classify multiclass MI tasks as introduced in Section 3.1. Since there are four classes, we can have four pairs of MI tasks: left vs rest (L/RE), right vs rest (R/RE), foot vs rest (F/RE), and tongue vs rest (T/RE). For each subject, the pair with the highest accuracy is used to train N.1, and the pair with the second highest accuracy is to train N.2.  Table 1 gives the ten-folder cross-validation results obtained using FGMDRM in OVR scheme.

Table 2 displays the kappa values obtained by method 1. Compared with other methods, five subjects (A03, A06, A07, A08, and A09) achieved higher kappa value of nine without exploring the frequency domain information by method 1. In the case of fixed frequency window, we have improved the mean kappa value of 0.069 than MDRM (*p*=0.4683), and 0.139 than FGMDM_fixed (*p*=0.1423). Our approach also shows significant improvement than FGMDM (*p*=0.6607), which has exploited subject-specific frequency information, in terms of the kappa value of 0.039.

### 5.2. Results of Method 2

**Figure 9:**
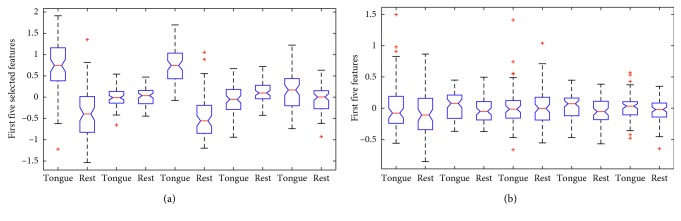
The box plot of five features of subject A09. (a) One-way ANOVA analysis on the first five features after applying *semi*-JMI. (b) One-way ANOVA analysis on the first five features from primitive vectors.

The results in Figure 9 show the T/RE feature distribution of the five features of subject A09.  Figure 9(a) shows the first five ranked features with *semi*-JMI. After applying the *semi*-JMI, the first five best features extracted have shown statistically significant improvement in the separability with *p* values <0.05 except feature 2 with *p* value 0.77. In Figure 9(b), the first five features extracted from primitive feature vectors with *p* value of 0.13, 0.05, 0.87, 0.05, and 0.13. The *p* values indicate that the pair T/RE have no significance in the primitive feature vectors. The results show that with our semisupervised feature ranking algorithm, the separable degree of the feature has been greatly improved.

**Figure 10:**
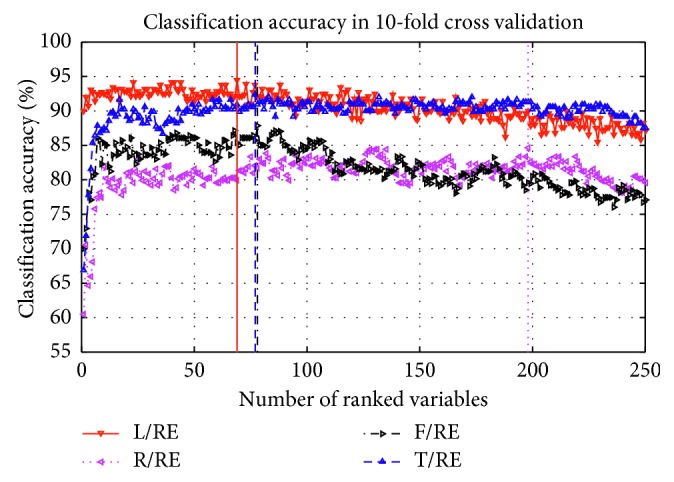
Classification accuracy corresponding the number of selected variables of Subject A09.

Figure 10 shows the evolution of the classification accuracy with KNN (*k* = 5 in this paper) against the number of ranked variables in OVR scheme. L/RE and T/RE are the two pairs with the highest recognition rate, and they achieved the highest recognition rate in 100 variables. But this is still a curse of dimensionality for classifiers; GDA is used to analyze the first 100 sorted variables in our study.

**Figure 11:**
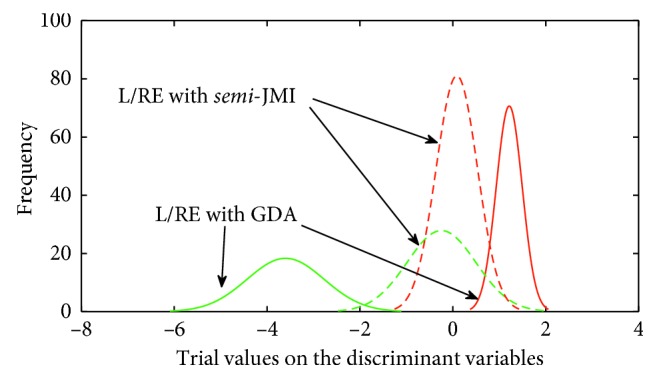
Feature distribution for the most discriminant variables in tangent space of Subject A09.

As the separation of characteristics cannot meet our requirements, GDA is used to get more obvious variables.  Figure 11 illustrates distributions for the first five most discriminant variables with GDA and *semi*-JMI. It can be seen from Figure 11 that L/RE is separated equally well by using GDA.

Table 3 displays ten-folder cross-validation results obtained using SJGDA and KNN in OVR scheme. It can be seen that the vectors which are mapped to the tangent space have better classification performance than that in the Riemannian manifold directly.

Table 4 presents the results obtained by SJGDA in pairwise way for multiclass MI tasks. We have six pairs of MI tasks: left and right (L/R), left and foot (L/F), left and tongue (L/T), right and foot (R/F), right and tongue (R/T), and foot and tongue (F/T).

Table 5 displays the comparison of classification accuracy using SJGDA and KNN for L/R task in 10-folder cross validation. References [8, 43–45] contain the classification of other publications. We have improved the accuracy compared with Reference [44] (*p* = 0.85) and Reference [45] (*p*=0.45). Gaur et al. [43] (*p*=0.95) explored the specific frequency information for each subject, and Sadatnejad and Shiry Ghidary [8] (*p*=0.90) used a novel kernel for dimensionality reduction which is similar to SJGDA. Although the results in the paper are not as high as those in Reference [43], it can be concluded that there is no difference between the results in Reference [43] and those in this paper because of *p*=0.95.

Table 6 presents the results in terms of the kappa value. The proposed method 1 achieved a mean performance of 0.589 which ranks this method to the first place of the competition. And with our proposed method 2, we have achieved a mean performance of 0.607, which makes method 2 to acquire the best performance of the state of the art.

Dataset 2 is used to verify the effect of method 2, and the classification results are given directly in this paper. The results are shown in Table 7. As can be seen from Table 7, method 2 obtained the second highest recognition rate in the comparative literature. Compared with the recent reference [47], method 2 achieved good classification results.

### 5.3. Results of Dataset 3

**Figure 12:**
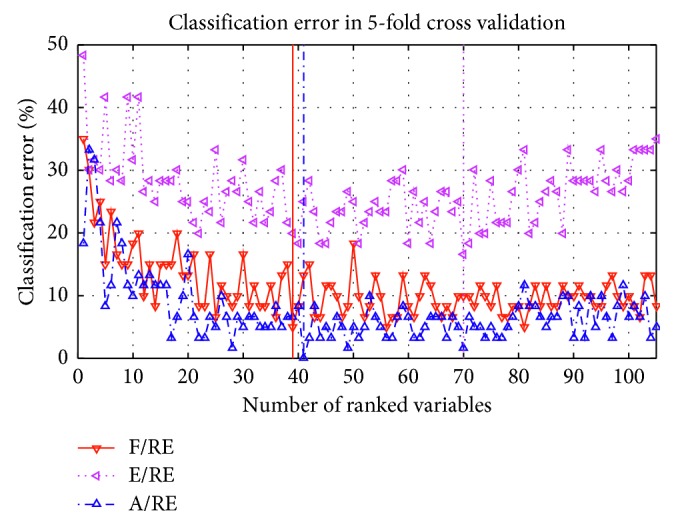
Classification accuracy corresponding to the number of selected variables of S01.

Dataset 3 is used to evaluate the performance of method 2.  Figure 12 shows the classification error with KNN against the number of ranked variables in OVR scheme. A/RE and F/RE are the two pairs with the lowest classification error, and they all achieved the highest recognition rate within 60 variables. In this paper, the first 60 ranked variables are used for the next analysis.

**Figure 13:**
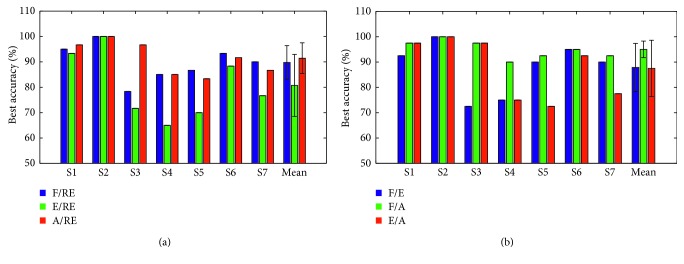
The comparison of three MI tasks in OVR scheme of seven subjects: (a) F/RE, E/RE, and A/RE; (b) F/E, F/A, and E/A.

Figure 13 displays 5-folder cross-validation results obtained by using SJGDA and KNN in OVR and OVO scheme. This Figure 13(a) illustrates three possible pairs of MI tasks (F/RE, E/RE, and A/RE) for each subject. It can be learned from the figure that flexion and abduction are the easiest movement to distinguish in six subjects of seven, and the six subjects are S1, S3, S4, S5, S6, and S7. However, due to individual differences, the highest recognition rate of each subject is different.

We also compared three possible pairs (F/E, F/A, and E/A) in OVO scheme of seven subjects.  Figure 13(b) depicts the comparison results for each subject, and it can be seen that the pair of F/A obtained the highest recognition rate in seven subjects. Combined with the analysis results of Figures 13(a) and 13(b), it can be considered that flexion and extension are more obvious in the three MI tasks.

**Figure 14:**
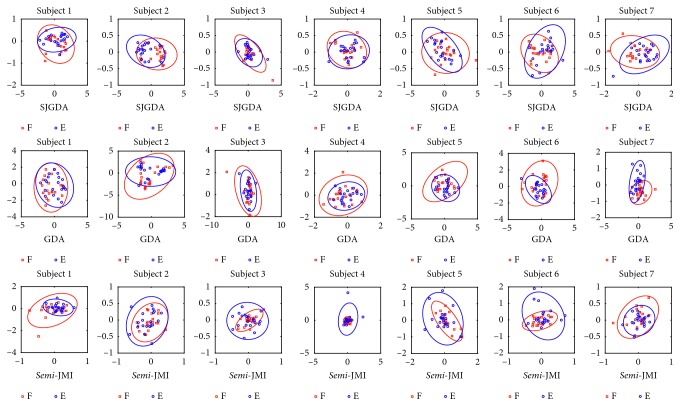
Feature distribution of F/E MI tasks extracted by SJGDA, GDA, and *semi*-JMI in dataset 2.

As SJGDA is a new method proposed in this paper, we also compared the feature distribution of SJGDA, GDA, and *semi*-JMI to illustrate the effectiveness of SJGDA.  Figure 14 depicts the feature distribution of F/E MI tasks of seven subjects. The blue and red circles represent the two different feature classes. As shown in Figure 14, the F/E MI tasks learned by SJGDA have high separability than GDA and *semi*-JMI.

**Figure 15:**
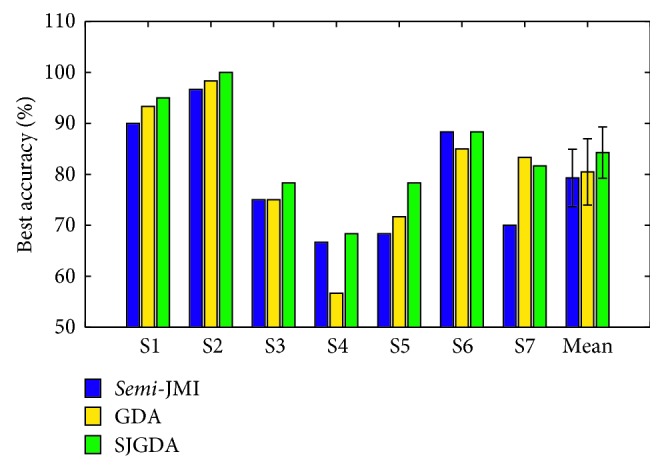
The comparison of the three different feature selection methods (*semi*-JMI, GDA, and SJGDA) with the same classifier (DT-KNN) of three MI tasks classification accuracy for each subject is depicted. The results are obtained by 5-folder cross validation.

The performance of the proposed method 2 is evaluated by using classification accuracy. Since there are three classes, the chance level is 33.33%.  Figure 15 demonstrates that the proposed method achieves higher performance for six subjects (S1, S2, S3, S4, S5, and S6) out of seven except S7 compared to *semi*-JMI and GDA methods. In addition, it also can be seen that GDA obtains a better classification accuracy for four subjects of seven (S1, S2, S5, and S7) compared with *semi*-JMI. The reasons for this phenomenon can be attributed to as follows: In the process of feature selection, we manually select feature dimensions suitable for classifiers, which results in partial information loss. As a feature dimensionality reduction technique, GDA is suitable for the preservation of useful information from the primitive vectors. And the proposed method SJGDA in this paper not only preserves the advantages of GDA but also adds some high ranking features to strengthen the expressive ability of the features.

## 6. Discussions

In this paper, we proposed a novel SSDT framework combined with classifiers to improve the performance of classifiers for multiclass MI tasks. We also proposed a novel NLDR method named SJGDA, and this NLDR method performs better than both *semi*-JMI and GDA on different datasets. In the following paragraphs, we have discussed the two methods in detail.

Method 1 indicates the drawback of FGMDRM, and then the novel SSDT framework is used to improve the accuracy for each individual. As shown in Table 2, compared with other published results, method 1 gets a quite good result in the case of processing the EEG signals of fixed frequency segment (8–30 Hz).

As shown in Table 6, Gaur et al. [43] proposed SS-MEMDBF to select the subject-specific frequency to obtain enhanced EEG signals which represent MI tasks related to *µ* and *β* rhythms, then classification with the Riemannian distance directly. TSLDA was proposed by Barachant et al. [10], and the covariance matrices are mapped onto a higher dimensional space where they can be vectorized and treated as Euclidean objects. Ang et al. [46] is the winner of the competition, FBCSP and multiple OVR classifiers were used for MI tasks, and achieved the mean kappa value of 0.57. Sadatnejad and Shiry Ghidary [8] proposed a new kernel for NLDR over the manifold of SPD matrices, the kappa value is 0.576. Davoudi et al. [14] considered the geometry of SPD matrices and provides a low-dimensional representation of the manifold with high-class discrimination, and the best result of this method in terms of the kappa value is 0.60.

In method 2, SJGDA is used to get more obvious vectors from the tangent vectors, and a SSDT-KNN classifier is used to identify different MI tasks. Combined with SJGDA and SSDT-KNN, we have achieved a better performance compared with method 1 (*p*=0.8632), Reference [43] (*p*=0.9586), TSLDA (*p*=0.6894), winner 1 (*p*=0.7051), Reference [8] (*p*=0.7245), and Reference [14] (*p*=0.9353). It is clear that the proposed method in this paper is effective for MI tasks in a BCI system.

In order to prove the effectiveness of the proposed method 2, we tested it on two other datasets. As shown in Table 7 and Figure 15, method 2 achieves good classification results on two datasets.

## 7. Conclusion

The experimental results of method 1 show that the proposed classification framework significantly improves the classification performance of the classifier. The experimental results of method 2 show that the SJGDA algorithm proposed in this paper is superior to GDA and *semi*-JMI in feature extraction, and method 2 has the highest recognition rate in this paper. However, as the classifiers in the SSDT framework is substitutable, the focus of the next work is to combine more advanced classifiers with SSDT to increase the recognition rate of the BCI systems.

## Figures and Tables

**Table 1:** Ten-folder cross-validation classification accuracy (%) for FGMDRM with OVR scheme applied on BCI competition dataset 2A.

Subject	A01	A02	A03	A04	A05	A06	A07	A08	A09	Mean	Std
L/RE	84.40	67.39	93.05	77.64	66.96	74.34	86.33	93.83	94.09	82.00	10.90
R/RE	**89.26**	75.33	**95.16**	78.56	63.92	71.81	81.18	93.39	82.92	81.28	10.24
F/RE	77.80	**83.44**	89.20	**80.88**	71.56	**78.22**	88.89	79.18	84.4	81.51	5.65
T/RE	88.21	68.76	90.59	79.95	**71.85**	76.72	**90.64**	**94.43**	**94.38**	83.95	9.82

**Table 2:** Kappa value comparison by SSDT-FGMDRM with other published results.

Subject	A01	A02	A03	A04	A05	A06	A07	A08	A09	Mean	*p* value
Method 1	0.66	0.39	**0.78**	0.47	0.25	**0.41**	**0.72**	**0.79**	**0.83**	**0.589**	
MDRM [10]	**0.75**	0.37	0.66	**0.53**	**0.29**	0.27	0.56	0.58	0.68	0.52	0.4683
FGMDM [14]	0.72	**0.50**	0.64	0.38	0.28	0.34	0.64	0.68	0.75	0.55	0.6607
FGMDM_fixed [14]	0.69	0.35	0.60	0.28	0.21	0.30	0.46	0.62	0.53	0.45	0.1423

**Table 3:** Ten-folder cross-validation results (%) obtained using SJGDA and KNN in OVR scheme applied on BCI competition dataset 2A.

Subject	A01	A02	A03	A04	A05	A06	A07	A08	A09	Mean	Std
L/RE	90.29	74.62	93.05	75.02	75.31	**78.15**	85.08	92.35	**95.14**	84.33	8.12
R/RE	**91.34**	77.11	**94.47**	77.07	73.61	75.74	81.58	93.41	87.53	83.54	7.75
F/RE	85.06	**83.70**	84.77	77.11	75.36	77.09	88.17	82.30	85.44	82.11	4.24
T/RE	88.89	76.72	89.25	**80.91**	**78.51**	76.04	**89.27**	**94.08**	93.41	85.23	6.76

**Table 4:** Ten-folder cross-validation results (%) obtained using SJGDA and KNN in OVO scheme applied on BCI competition dataset 2A.

Subject	A01	A02	A03	A04	A05	A06	A07	A08	A09	Mean	Std
L/R	90.95	67.29	94.52	63.87	64.48	70.33	70.29	**97.95**	95.00	79.41	13.85
L/F	95.19	87.43	93.93	81.84	68.10	**77.14**	**98.57**	88.29	93.81	87.14	9.29
L/T	96.52	64.24	**96.57**	83.24	**73.67**	69.91	97.95	97.23	**99.29**	86.51	13.16
R/F	95.90	**87.52**	95.05	**84.84**	66.71	72.88	97.24	93.18	87.43	86.75	10.01
R/T	**99.33**	79.82	96.48	79.12	72.22	71.48	97.24	95.05	92.29	87.00	10.61
F/T	83.94	84.17	86.62	75.10	62.62	74.21	88.31	93.10	90.33	82.04	9.10

**Table 5:** Comparison of classification accuracy (%) for L/R task with other published results using OVO scheme applied on BCI competition dataset 2A.

Subject	A01	A02	A03	A04	A05	A06	A07	A08	A09	Mean	Std	*p* value
Method 2	90.95	**67.29**	94.52	63.87	**64.48**	70.33	70.29	**97.95**	**95.00**	79.41	13.85	
Reference [43]	**91.49**	60.56	94.16	76.72	58.52	68.52	78.57	97.01	93.85	**79.93**	14.13	0.95
Reference [8]	88.89	59.03	90.28	**78.47**	62.50	**75.00**	72.92	93.06	87.50	78.63	11.63	0.90
Reference [44]	88.89	51.39	**96.53**	70.14	54.86	71.53	**81.25**	93.75	93.75	78.01	16.04	0.85
Reference [45]	90.28	54.17	93.75	64.58	57.64	65.28	62.50	90.97	85.42	73.84	15.02	0.45

**Table 6:** Kappa value comparison with other published results.

Subject	A01	A02	A03	A04	A05	A06	A07	A08	A09	Mean	*p* value
Method 2	0.77	0.38	0.76	0.47	0.27	**0.42**	0.73	**0.81**	**0.85**	**0.607**	
Method 1	0.66	0.39	**0.78**	0.47	0.25	0.41	0.72	0.79	0.83	0.589	0.8632
Reference [43]	**0.86**	0.24	0.70	**0.68**	0.36	0.34	0.66	0.75	0.82	0.60	0.9586
TSLDA [10]	0.74	0.38	0.72	0.50	0.26	0.34	0.69	0.71	0.76	0.567	0.6894
Winner 1 [46]	0.68	0.42	0.75	0.48	**0.40**	0.27	0.77	0.75	0.61	0.57	0.7051
Reference [8]	0.71	0.46	0.76	0.44	0.26	0.37	**0.79**	0.75	0.61	0.57	0.7245
Reference [14]	0.75	**0.49**	0.76	0.49	0.34	0.36	0.68	0.76	0.76	0.60	0.9353

**Table 7:** Five-folder cross validation by method 2 applied on BCI III dataset IIIa.

Subject	Method 2	Reference [47]	Reference [48]	Reference [49]	Reference [50]
k3b	91.67	90.00	86.67	94.20	**94.44**
k6b	75.00	76.25	**81.67**	69.00	62.50
l1b	81.67	77.91	**85.00**	78.60	78.33
Mean	82.78	81.38	**84.44**	80.60	78.42
*p* value		0.96	0.42	0.94	0.73

## Data Availability

The dataset 1 and dataset 2 used to support the findings of this study are available from http://bnci-horizon-2020.eu/database/data-sets. The dataset 3 used to support the findings of this study is available from the corresponding author upon request.

## References

[1] Wolpaw J. R., Birbaumer N., McFarland D. J., Pfurtscheller G., Vaughan T. M. (2002). Brain-computer interfaces for communication and control. *Clinical neurophysiology*.

[2] Birbaumer N. (2006). Breaking the silence: brain?computer interfaces (BCI) for communication and motor control. *Psychophysiology*.

[3] Ang K. K., Guan C., Phua K. S. Transcranial direct current stimulation and EEG-based motor imagery BCI for upper limb stroke rehabilitation.

[4] Xu B., Peng S., Song A., Yang R., Pan L. (2011). Robot-aided upper-limb rehabilitation based on motor imagery EEG. *International Journal of Advanced Robotic Systems*.

[5] Wang F., Zhang X., Fu R., Sun G. (2018). Study of the home-auxiliary robot based on BCI. *Sensors*.

[6] Wang F., Wang H., Fu R. (2018). Real-Time ECG-based detection of fatigue driving using sample entropy. *Entropy*.

[7] Koles Z. J., Lazar M. S., Zhou S. Z. (1990). Spatial patterns underlying population differences in the background EEG. *Brain Topography*.

[8] Sadatnejad K., Shiry Ghidary S. (2016). Kernel learning over the manifold of symmetric positive definite matrices for dimensionality reduction in a BCI application. *Neurocomputing*.

[9] Congedo M., Barachant A., Bhatia R. (2017). Riemannian geometry for EEG-based brain-computer interfaces; a primer and a review. *Brain-Computer Interfaces*.

[10] Barachant A., Bonnet S., Congedo M., Jutten C. (2012). Multiclass brain-computer interface classification by riemannian geometry. *IEEE Transactions on Biomedical Engineering*.

[11] Barachant A., Bonnet S., Congedo M., Jutten C. Common spatial pattern revisited by riemannian geometry.

[12] Barachant A., Bonnet S., Congedo M., Jutten C. A brain-switch using riemannian geometry.

[13] Barachant A., Bonnet S., Congedo M., Jutten C. (2013). Classification of covariance matrices using a riemannian-based kernel for BCI applications. *Neurocomputing*.

[14] Davoudi A., Ghidary S. S., Sadatnejad K. (2016). Dimensionality reduction based on distance preservation to local mean (DPLM) for spd matrices and its application in BCI. https://arxiv.org/abs/1608.00514.

[15] Brandl S., Müller K.-R., Samek W. Robust common spatial patterns based on Bhattacharyya distance and gamma divergence.

[16] Samek W., Kawanabe M., Muller K.-R. (2014). Divergence-based framework for common spatial patterns algorithms. *IEEE Reviews in Biomedical Engineering*.

[17] Samek W., Müller K.-R. Information geometry meets BCI spatial filtering using divergences.

[18] Samek W., Kawanabe M. Robust common spatial patterns by minimum divergence covariance estimator.

[19] Kumar S., Mamun K., Sharma A. (2017). CSP-TSM: optimizing the performance of riemannian tangent space mapping using common spatial pattern for mi-BCI. *Computers in biology and medicine*.

[20] Lindig-León C., Gayraud N., Bougrain L., Clerc M. Comparison of hierarchical and non-hierarchical classification for motor imagery based BCI systems.

[21] Roweis S. T., Saul L. K. (2000). Nonlinear dimensionality reduction by locally linear embedding. *Science*.

[22] Kramer O., Lückehe D. Visualization of evolutionary runs with isometric mapping.

[23] Weinberger K. Q., Sha F., Saul L. K. Learning a kernel matrix for nonlinear dimensionality reduction.

[24] Van Der Maaten L. (2009). Learning a parametric embedding by preserving local structure. *RBM*.

[25] Van Der Maaten L. (2014). Accelerating t-SNE using tree-based algorithms. *Journal of Machine Learning Research*.

[26] Lee D., Park S.-H., Lee S.-G. (2017). Improving the accuracy and training speed of motor imagery brain-computer interfaces using wavelet-based combined feature vectors and Gaussian mixture model-supervectors. *Sensors*.

[27] Xie X., Yu Z. L., Lu H., Gu Z., Li Y. (2017). Motor imagery classification based on bilinear sub-manifold learning of symmetric positive-definite matrices. *IEEE Transactions on Neural Systems and Rehabilitation Engineering*.

[28] Harandi M. T., Salzmann M., Hartley R. From manifold to manifold: Geometry-aware dimensionality reduction for SPD matrices.

[29] Moakher M. (2005). A differential geometric approach to the geometric mean of symmetric positive-definite matrices. *SIAM Journal on Matrix Analysis and Applications*.

[30] Fletcher P. T., Joshi S., Sonka M., Kakadiaris I. A., Kybic J. (2004). Principal geodesic analysis on symmetric spaces: statistics of diffusion tensors. *Computer Vision and Mathematical Methods in Medical and Biomedical Image Analysis*.

[31] Barachant A., Bonnet S., Congedo M., Jutten C. Riemannian geometry applied to BCI classification.

[32] Guo H., Gelfand S. B. (1992). Classification trees with neural network feature extraction. *IEEE Transactions on Neural Networks*.

[33] Safavian S. R., Landgrebe D. (1991). A survey of decision tree classifier methodology. *IEEE Transactions on Systems, Man, and Cybernetics*.

[34] Shao Y.-H., Chen W.-J., Huang W.-B., Yang Z.-M., Deng N.-Y. (2013). The best separating decision tree twin support vector machine for multi-class classification. *Procedia Computer Science*.

[35] Brown G., Pocock A., Zhao M.-J., Luján M. (2012). Conditional likelihood maximisation: a unifying framework for information theoretic feature selection. *Journal of Machine Learning Research*.

[36] Sechidis K., Brown G. (2017). Simple strategies for semi-supervised feature selection. *Machine Learning*.

[37] Moreno-Torres J. G., Raeder T., Alaiz-Rodríguez R., Chawla N. V., Herrera F. (2012). A unifying view on dataset shift in classification. *Pattern Recognition*.

[38] Baudat G., Anouar F. (2000). Generalized discriminant analysis using a kernel approach. *Neural Computation*.

[39] Haghighat M., Zonouz S., Abdel-Mottaleb M. (2015). Cloudid: trustworthy cloud-based and cross-enterprise biometric identification. *Expert Systems with Applications*.

[40] Vapnik V. (2013). *The Nature of Statistical Learning Theory*.

[41] Brunner C., Leeb R., Müller-Putz G., Schlögl A., Pfurtscheller G. (2008). *BCI Competition 2008–Graz Data Set A*.

[42] Blankertz B., Muller K. R., Krusienski D. J. (2006). The BCI competition III: validating alternative approaches to actual BCI problems. *IEEE Transactions on Neural Systems and Rehabilitation Engineering*.

[43] Gaur P., Pachori R. B., Wang H., Prasad G. (2018). A multi-class EEG-based BCI classification using multivariate empirical mode decomposition based filtering and riemannian geometry. *Expert Systems with Applications*.

[44] Lotte F., Cuntai Guan C. (2011). Regularizing common spatial patterns to improve BCI designs: unified theory and new algorithms. *IEEE Transactions on Biomedical Engineering*.

[45] Raza H., Cecotti H., Li Y., Prasad G. (2015). Adaptive learning with covariate shift-detection for motor imagery-based brain-computer interface. *Soft Computing*.

[46] Ang K. K., Chin Z. Y., Wang C., Guan C., Zhang H. (2012). Filter bank common spatial pattern algorithm on BCI competition IV datasets 2a and 2b. *Frontiers in Neuroscience*.

[47] Baali H., Khorshidtalab A., Mesbah M., Salami M. J. E. (2015). A transform-based feature extraction approach for motor imagery tasks classification. *IEEE Journal of Translational Engineering in Health and Medicine*.

[48] Schlögl A., Lee F., Bischof H., Pfurtscheller G. (2005). Characterization of four-class motor imagery EEG data for the BCI-competition 2005. *Journal of Neural Engineering*.

[49] Grosse-Wentrup M., Buss M. (2008). Multiclass common spatial patterns and information theoretic feature extraction. *IEEE Transactions on Biomedical Engineering*.

[50] Koprinska I. Feature selection for brain-computer interfaces.

